# Supplementary material for: The causal role of circulating inflammatory markers in osteoporosis: a bidirectional Mendelian randomized study
Source: Front Immunol. 2024 Jul 18;15:1412298. doi: 10.3389/fimmu.2024.1412298 (PMC11291241; doi:10.3389/fimmu.2024.1412298)
Supplement: Supplementary file 1 [file DataSheet_1.zip › supplementary material/OSTEOPOROSIS-CIM/GCST90274780.tsv.gz_leave_one_out.pdf]

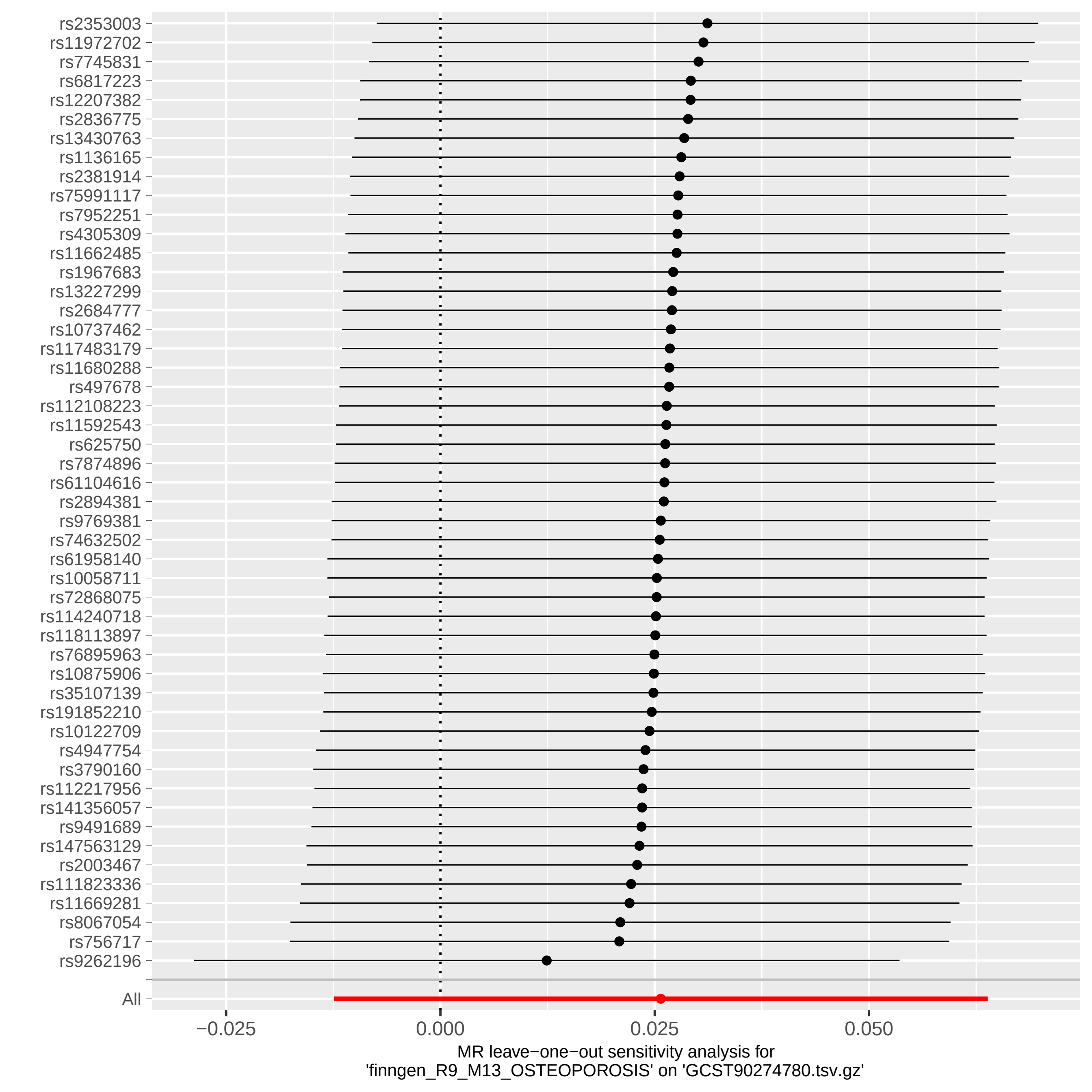
rs2353003
rs11972702
rs7745831
rs6817223
rs12207382
rs2836775
rs13430763
rs1136165
rs2381914
rs75991117
rs7952251
rs4305309
rs11662485
rs1967683
rs13227299
rs2684777
rs10737462
rs117483179
rs11680288
rs497678
rs112108223
rs11592543
rs625750
rs7874896
rs61104616
rs2894381
rs9769381
rs74632502
rs61958140
rs10058711
rs72868075
rs114240718
rs118113897
rs76895963
rs10875906
rs35107139
rs191852210
rs10122709
rs4947754
rs3790160
rs112217956
rs141356057
rs9491689
rs147563129
rs2003467
rs111823336
rs11669281
rs8067054
rs756717
rs9262196
All
−0.025
0.000
0.025
0.050
MR leave−one−out sensitivity analysis for
'finngen_R9_M13_OSTEOPOROSIS' on 'GCST90274780.tsv.gz'
